# *Bifidobacterium pseudocatenulatum* LI09 and *Bifidobacterium catenulatum* LI10 attenuate D-galactosamine-induced liver injury by modifying the gut microbiota

**DOI:** 10.1038/s41598-017-09395-8

**Published:** 2017-08-18

**Authors:** Daiqiong Fang, Ding Shi, Longxian Lv, Silan Gu, Wenrui Wu, Yanfei Chen, Jing Guo, Ang Li, Xinjun Hu, Feifei Guo, Jianzhong Ye, Yating Li, Lanjian Li

**Affiliations:** 1 State Key Laboratory for Diagnosis and Treatment of Infectious Diseases, The First Affiliated Hospital, College of Medicine, Zhejiang University, Hangzhou, Zhejiang, 310003 China; 2 Collaborative Innovation Center for Diagnosis and Treatment of Infectious Diseases, Hangzhou, 310003 China

## Abstract

The gut microbiota is altered in liver diseases, and several probiotics have been shown to reduce the degree of liver damage. We hypothesized that oral administration of specific *Bifidobacterium* strains isolated from healthy guts could attenuate liver injury. Five strains were tested in this study. Acute liver injury was induced by D-galactosamine after pretreating Sprague-Dawley rats with the *Bifidobacterium* strains, and liver function, liver and ileum histology, plasma cytokines, bacterial translocation and the gut microbiome were assessed. Two strains, *Bifidobacterium pseudocatenulatum* LI09 and *Bifidobacterium catenulatum* LI10, conferred liver protection, as well as alleviated the increase in plasma M-CSF, MIP-1α and MCP-1 and bacterial translocation. They also ameliorated ileal mucosal injury and gut flora dysbiosis, especially the enrichment of the opportunistic pathogen *Parasutterella* and the depletion of the SCFA-producing bacteria *Anaerostipes*, *Coprococcus* and *Clostridium XI*. Negative correlations were found between MIP-1α / MCP-1 and *Odoribacter* (LI09 group) and MIP-1α / M-CSF and *Flavonifractor* (LI10 group). Our results indicate that the liver protection effects might be mediated through gut microbiota modification, which thus affect the host immune profile. The desirable characteristics of these two strains may enable them to serve as potential probiotics for the prevention or adjuvant treatment of liver injury.

## Introduction

Accumulating evidence shows that alterations in the intestinal microbiota play an important role in the progression of liver injury^1–3^. The liver interacts directly with the intestine through the portal system and the bile secretion system^4^. Under normal circumstances, a series of local and systemic protective mechanisms, including intestinal colonization resistance, can prevent the passage of potentially pathogenic bacteria or bacterial components, such as lipopolysaccharide, beyond the intestinal lumen^5, 6^. When the normal liver physiology is disrupted, followed by the weakening of the gut barrier, the movement of these microbes and their products from the intestinal lumen to the liver is likely to aggravate certain liver diseases by enhancing the propagation of inflammation and tissue damage, and spontaneous bacterial peritonitis could even occur^7^. Thus, a therapy aimed at preserving the intestinal colonization resistance by modulating the gut flora, may aid in the prevention or adjuvant treatment of hepatic injury.

A previous study showed that the *Bifidobacterium*/*Enterobacteriaceae* (B/E) ratio, which may indicate the microbial colonization resistance of the bowel, decreased significantly in patients with liver diseases^8^. *Bifidobacterium* species are generally used as probiotics because of their associated health-promoting benefits and “GRAS” (generally recognized as safe) status^9^. *Bifidobacterium longum*, a major ingredient of VSL#3 (VSL#3 is a high potency probiotic medical food designated for ulcerative colitis, irritable bowel syndrome, etc. and it contains 8 diverse strains of bacteria), and *Bifidobacterium pseudocatenulatum* can potentially attenuate cirrhosis^10–14^, and *Bifidobacterium catenulatum* has been proven to be an effective treatment for acute liver injury^15, 16^. However, the definitive mechanism by which *Bifidobacterium* strains protect against liver injury and their influence on modulation of gut microbiota composition have not been completely explored in previous studies.

In this study, we examined the effects of *Bifidobacterium longum* LI06 (CGMCC 10385), *Bifidobacterium longum* LI07 (CGMCC 10386), *Bifidobacterium pseudocatenulatum* LI08 (CGMCC 10387), *Bifidobacterium pseudocatenulatum* LI09 (CGMCC 10388) and *Bifidobacterium catenulatum* LI10 (CGMCC 10389) on acute liver injury induced by D-galactosamine (D-GalN). Furthermore, we explored the relevant mechanism by which these potential probiotic strains protect against liver injury by examining plasma inflammatory cytokines, terminal ileum histology, bacterial translocation and the gut flora composition. Our results showed that *B. pseudocatenulatum* LI09 and *B. catenulatum* LI10 could attenuate D-GalN induced liver damage, as well as systemic inflammatory responses, bacterial translocation, ileal mucosal injury and gut flora dysbiosis, with gut microbiota modification.

## Results

### *Bifidobacterium pseudocatenulatum* LI09 and *Bifidobacterium catenulatum* LI10 alleviated the D-GalN-induced liver injury

Twenty-four hours after the induction of liver damage, none of the rats had died. To examine the effects of the *Bifidobacterium* strains on D-GalN-induced acute liver injury, we first compared the liver function of *Bifidobacterium*-treated rats with that of positive acute liver injury control (PC) rats. Among the five *Bifidobacterium* strains used in this study, oral administration with *B. longum* LI06, *B. pseudocatenulatum* LI08, *B. pseudocatenulatum* LI09 and *B. catenulatum* LI10 significantly reduced the D-GalN-induced increase in alanine aminotransferase (ALT) and total bile acid, but *B. longum LI07* did not (Table 1). The increase in glutamyltransferase induced by D-GalN was significantly reduced by *B. longum* LI06, *B. pseudocatenulatum* LI08 and *B. catenulatum* LI10. In addition, only administration with *B. pseudocatenulatum* LI09 and *B. catenulatum* LI10 relieved the increase in aspartate aminotransferase (AST) and glycylproline dipeptidyl aminopeptidase. Therefore, *B. pseudocatenulatum* LI09 and *B. catenulatum* LI10 seemed to more efficiently ameliorate the destruction of liver function induced by D-GalN.

**Table 1:** Effects of pretreatment with five *Bifidobacterium* strains on liver function during D-GalN-induced acute liver injury.

	ALT (U/L)	AST (U/L)	GGT (U/L)	GPDA (U/L)	TBA (µmol/L)	TB (µmol/L)	ALB (g/L)
LI06 (n = 9)	5005.0 ± 4147.8**	7505.0 ± 6705.4	10.9 ± 8.4*	303.6 ± 161.9	261.6 ± 167.0*	12.5 ± 10.3	37.2 ± 3.1
LI07 (n = 9)	6340.0 ± 5733.0	9135.0 ± 7725.8	14.6 ± 13.1	318.8 ± 203.9	284.6 ± 192.6	43.6 ± 83.7	36.7 ± 2.9
LI08 (n = 9)	6431.1 ± 2933.8*	8200.0 ± 4038.7	13.2 ± 6.6*	356.3 ± 135.3	326.0 ± 116.4*	23.7 ± 12.5	36.8 ± 1.5
LI09 (n = 9)	4191.1 ± 2777.8***	5800.0 ± 3601.0**	13.8 ± 11.4	272.4 ± 137.0**	285.2 ± 136.9*	17.4 ± 12.8	36.8 ± 1.7
LI10 (n = 9)	3888.9 ± 3190.7***	5471.1 ± 4247.0**	10.8 ± 8.2*	250.2 ± 151.0**	274.6 ± 177.0*	16.3 ± 12.5	37.1 ± 2.1
PC (n = 8)	10000.0 ± 2058.1	12050.0 ± 3342.2	21.5 ± 9.0	427.4 ± 56.3	440.1 ± 60.6	68.6 ± 88.0	34.5 ± 3.7
NC (n = 6)	49.5 ± 7.8***	101.2 ± 12.2***	NA	65.7 ± 14.1***	54.2 ± 24.3***	1.8 ± 0.4	37.3 ± 2.4

Values are expressed as the mean ± SD. The serum levels of aspartate aminotransferase (AST), alanine aminotransferase (ALT), glutamyltransferase (GGT), glycylproline dipeptidyl aminopeptidase (GPDA), total bile acid (TBA), total bilirubin (TB) and albumin (ALB) were determined after 24 h of D-galactosamine (D-GalN) administration. Compared with the PC group, *p < 0.05, **p < 0.01, ***p < 0.001.

**Figure 1:**
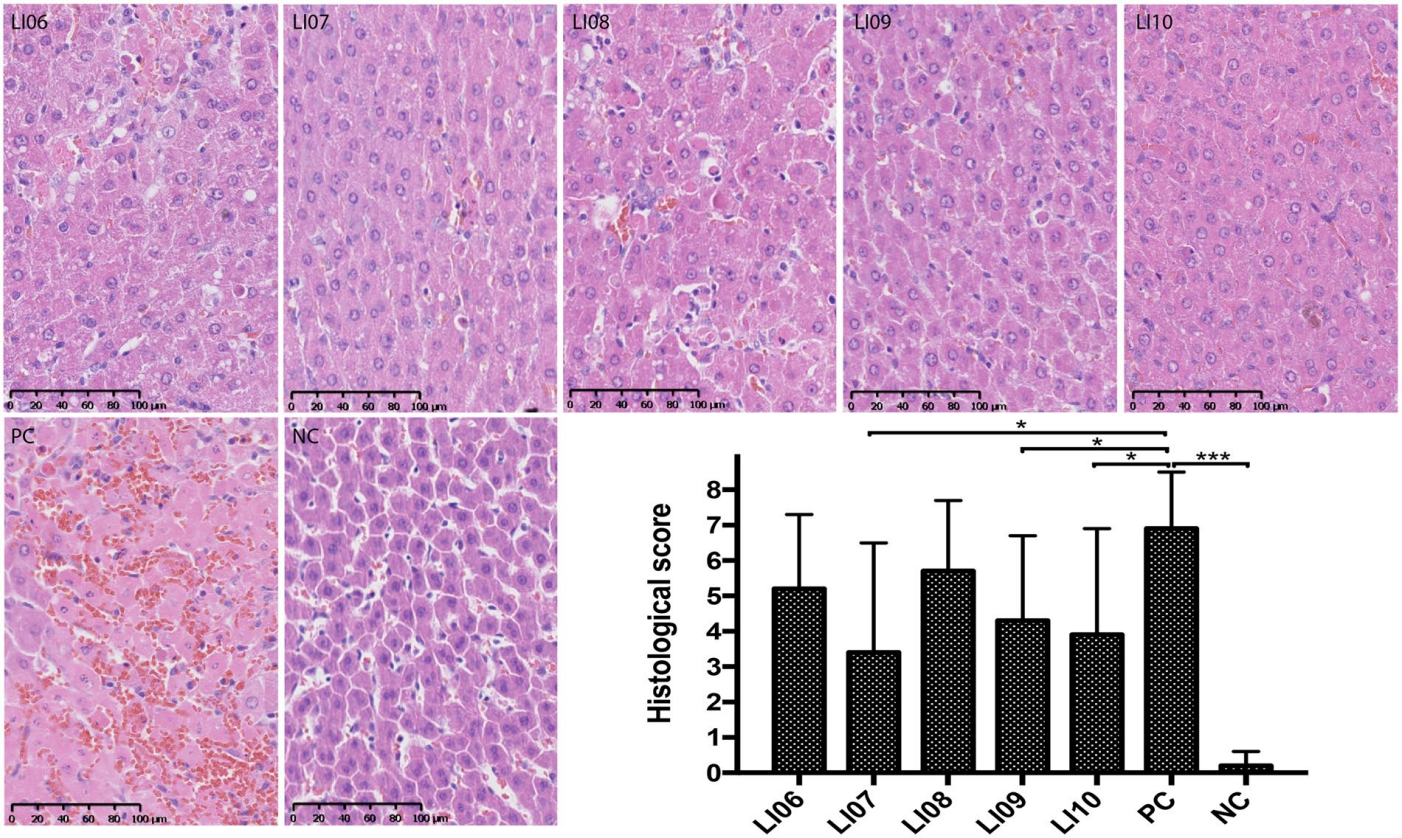
Effects of pretreatment with five *Bifidobacterium* strains on hepatic histological abnormalities during D-galactosamine (D-GalN)-induced acute liver injury. Representative images of hepatic haematoxylin and eosin (H&E) staining and histological scores of livers based on these images. Values are expressed as the mean ± SD. *p < 0.05, ***p < 0.001 compared with the positive acute liver injury control (PC) group.

To further confirm the effects of these *Bifidobacterium* strains on liver injury, we examined the liver morphology of rats in different groups. Twenty-four hours after the induction of liver injury, all groups, except the negative acute liver injury control (NC) group, demonstrated hepatic histological abnormalities (Fig. 1). Administration of *B. longum* LI07, *B. pseudocatenulatum* LI09 and *B. catenulatum* LI10 significantly ameliorated D-GalN-induced hepatic degeneration or necrosis and inflammatory cell infiltration. The results from two aspects, liver function and liver histology, both indicate the protective effects of *B. pseudocatenulatum* LI09 and *B. catenulatum* LI10 against hepatic injury.

### *B. pseudocatenulatum* LI09 and *B. catenulatum* LI10 reduced the D-GalN-induced increase in plasma inflammatory cytokines

**Figure 2:**
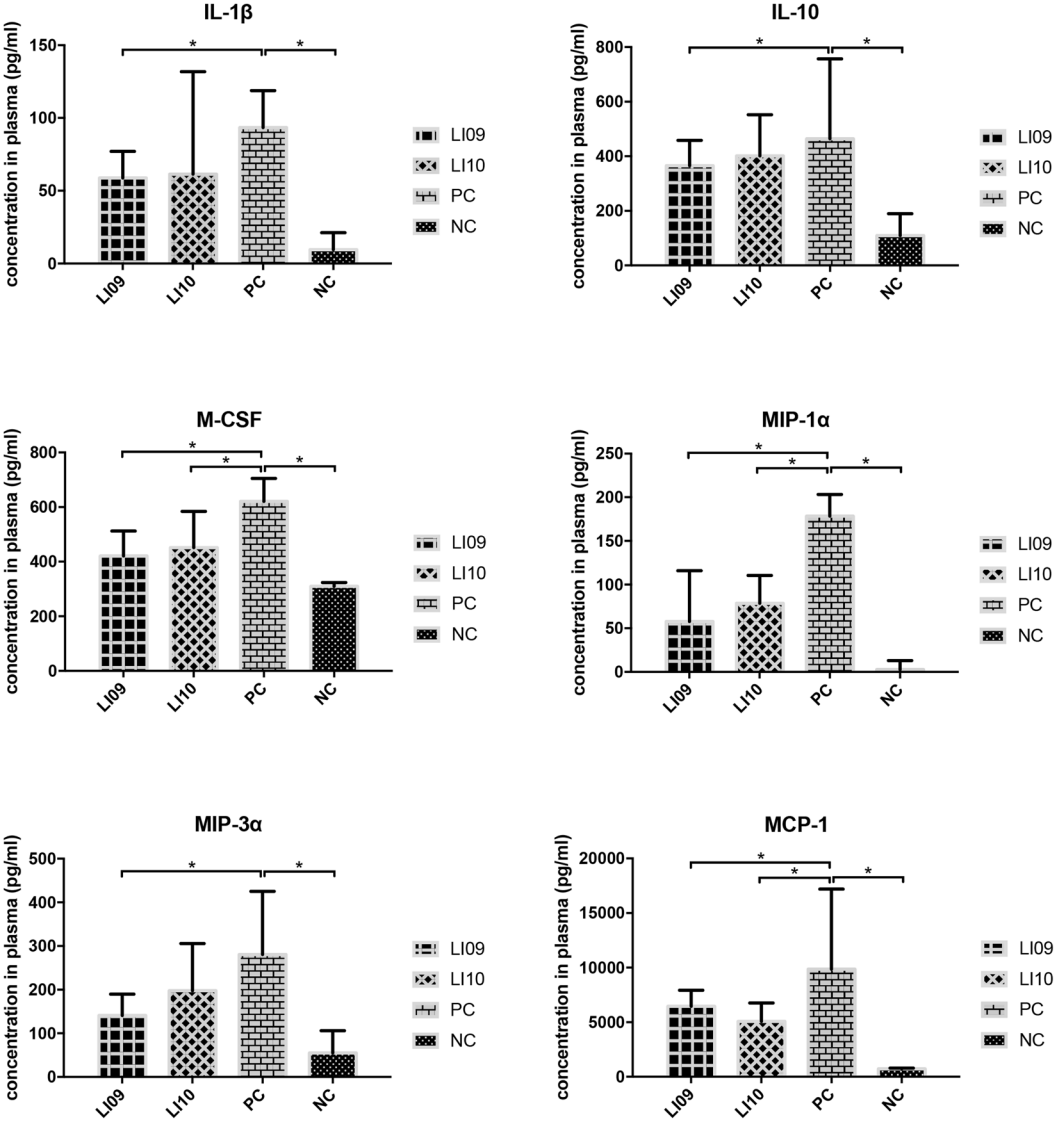
Effects of pretreatment with *B. pseudocatenulatum* LI09 or *B. catenulatum* LI10 on hypercytokinemia during D-GalN-induced acute liver injury. Values are expressed as the median with interquartile range. *p < 0.05 compared with the PC group.

Since liver injury is aggravated by inflammatory molecules, we next examined whether these two strains could relieve inflammation by measuring a total of 24 different plasma cytokines. Twenty-four hours after D-GalN treatment, the plasma levels of all detected cytokines increased in the PC group compared with those in the NC group. Administration of *B. pseudocatenulatum* LI09 significantly alleviated the D-GalN-induced increase in the proinflammatory cytokine interleukin (IL)-1β and the immunomodulatory cytokine IL-10 (Fig. 2). Among the chemokines, the increase in macrophage inflammatory protein 1 alpha (MIP-1α) and monocyte chemoattractant protein 1 (MCP-1) was attenuated when the rats were treated with either *B. pseudocatenulatum* LI09 or *B. catenulatum* LI10. However, the increase in another chemokine, MIP-3α, was only ameliorated in *B. pseudocatenulatum* LI09-treated rats. In addition, the increase in macrophage colony-stimulating factor (M-CSF) was also alleviated in the groups treated with these two strains. These results suggest that the reduction of chemokine levels may play a pivotal role in the process by which *Bifidobacterium* improves liver injury. By comparison, Administration of none of the *B. longum* LI06, *B. longum* LI07 or *B. pseudocatenulatum* LI08 could alleviate the increase of all these chemokines simultaneously (see Supplementary Fig. S1).

### *B. pseudocatenulatum* LI09 and *B. catenulatum* LI10 ameliorated the D-GalN-induced bacterial translocation

To determine how the inflammatory reaction could be attenuated, we investigated the gut barrier function, since oral administration of *Bifidobacterium* strains first and directly impacts gut. We examined the bacterial translocation to evaluate the protective effects of these two strains on D-GalN-induced destruction of the gut barrier. As shown in Table 2, administration of either *B. pseudocatenulatum* LI09 or *B. catenulatum* LI10 reduced the population of bacteria that translocated to mesenteric lymph nodes (MLNs), indicating the restorative effects of these two strains on gut barrier function. By contrast, administration of none of the *B. longum* LI06, *B. longum* LI07 or *B. pseudocatenulatum* LI08 could cut down the population of bacteria which translocated to MLNs (see Supplementary Table S1).

**Table 2:** Effects of pretreatment with *B. pseudocatenulatum* LI09 or *B. catenulatum* LI10 on bacterial translocation during D-GalN-induced acute liver injury.

	MLN (log10 CFU/g)
LI09 (n = 9)	2.0 ± 1.4*
LI10 (n = 9)	2.3 ± 1.2*
PC (n = 8)	3.7 ± 0.5
NC (n = 6)	2.8 ± 0.3*

Values are expressed as the mean ± SD. *p < 0.05 compared with the PC group.

### *B. pseudocatenulatum* LI09 and *B. catenulatum* LI10 attenuated the D-GalN-induced terminal ileum injury

**Figure 3:**
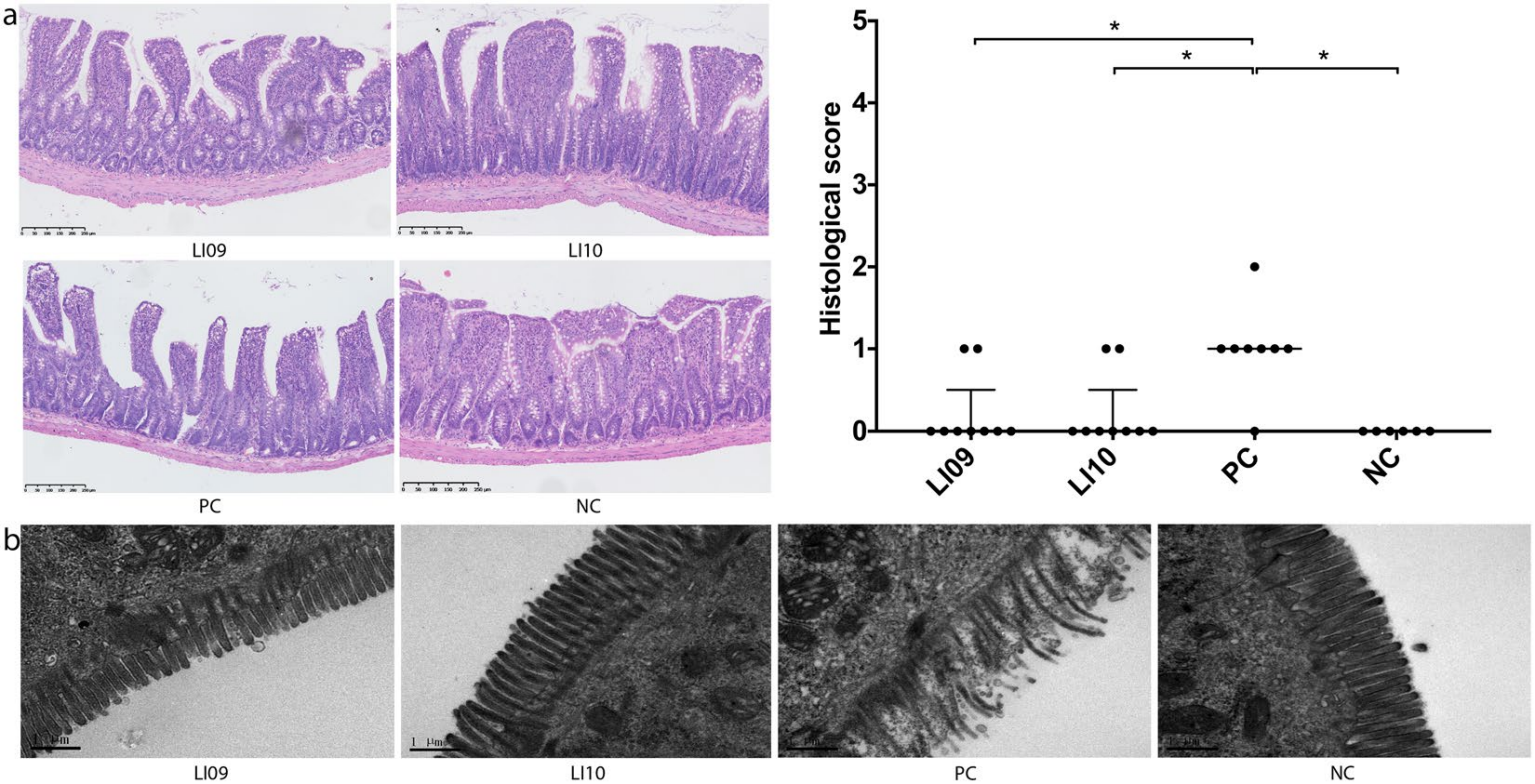
Effects of pretreatment with *B. pseudocatenulatum* LI09 or *B. catenulatum* LI10 on ileal histological abnormalities during D-GalN-induced acute liver injury. (**a**) Representative images of distal ileal H&E staining and histological scores of distal ileums based on these images. Values are expressed as the median with interquartile range. *p < 0.05 compared with the PC group. (**b**) Representative electron microscopy images of the distal ileum. Microvilli of the intestinal epithelial cells were examined.

Since the integrity of the intestinal mucosa is an important structure that supports the gut barrier function, we explored whether the intestinal mucosa integrity was damaged during D-GalN-induced liver injury and whether administering these strains improved the condition. As evaluated by haematoxylin and eosin (H&E) staining^17^, pretreatment with *B. pseudocatenulatum* LI09 or *B. catenulatum* LI10 alleviated the D-GalN-induced histological abnormalities of the terminal ileum by decreasing the incidence of subepithelial Gruenhagen’s space and epithelial lifting (Fig. 3a). In comparison, pretreatment with none of the *B. longum* LI06, *B. longum* LI07 or *B. pseudocatenulatum* LI08 could alleviate the histological abnormalities significantly (see Supplementary Fig. S2). We further observed the intestinal mucosal ultrastructure by transmission electron microscopy. Pretreatment with these two strains improved the ruptured, sparse, and stunted phenotypes of the intestinal epithelial cell microvilli (Fig. 3b).

### *B. pseudocatenulatum* LI09 and *B. catenulatum* LI10 alleviated the D-GalN-induced alterations of the gut microbiome

Since the normal flora acts as another form of intestinal barrier, and the ecological balance of the gut flora is disrupted in D-GalN-induced hepatic injury, we next explored how the gut microbiota changed upon the administration of these strains during D-GalN-induced liver injury by metagenomic sequencing of the bacterial 16 S rRNA V3–V4 regions. From 59 rat caecum content samples, 1 489 572 qualified reads were filtered for downstream analysis. A total of 5000 reads were randomly chosen from each sample to equilibrate the sequencing depth. Based on ≥97% sequence identity, 497 qualified operational taxonomic units (OTUs) were clustered. Four OTUs were discarded because of their low coverage (fewer than 5 samples).

**Figure 4:**
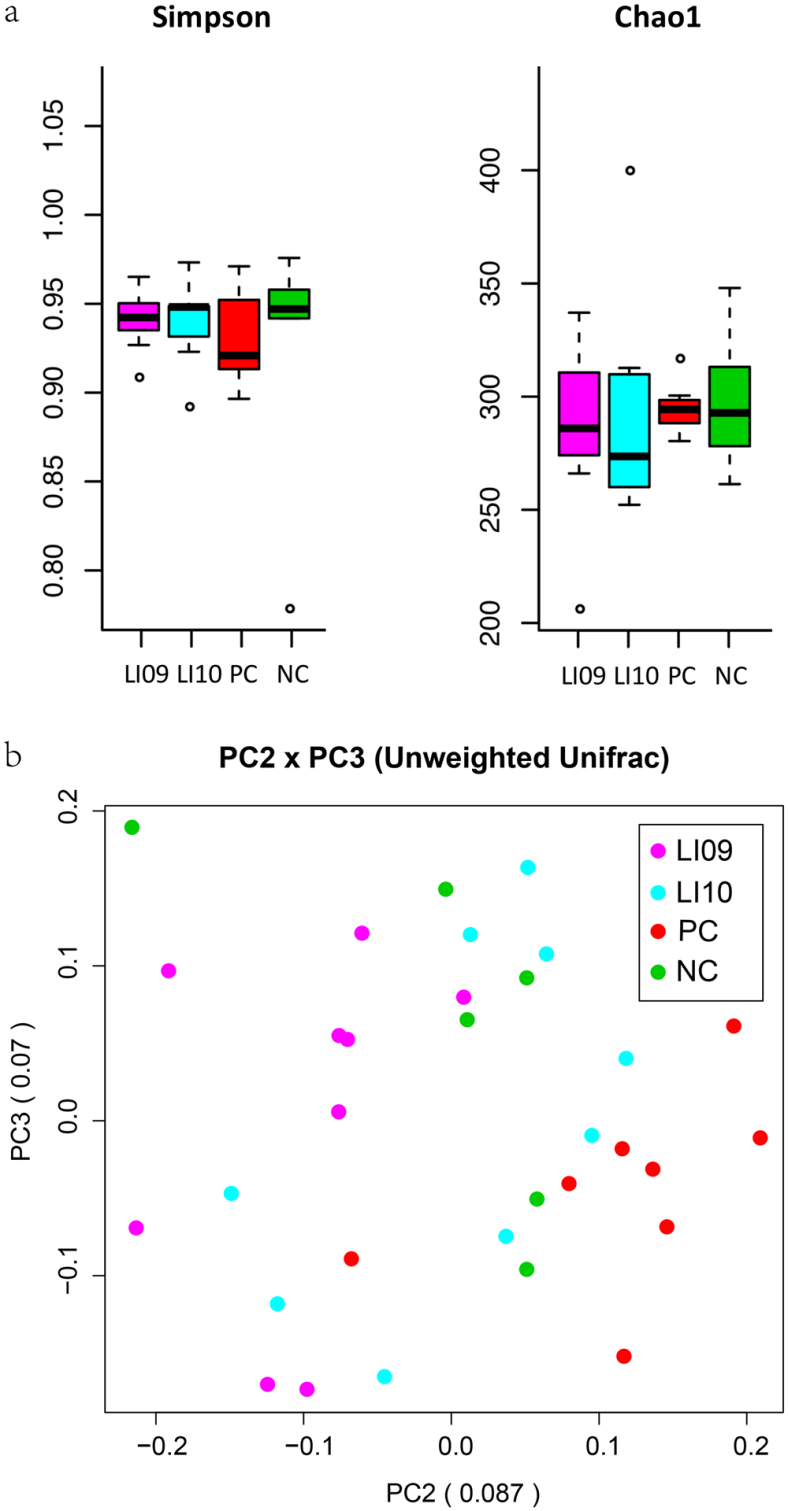
Effects of pretreatment with *B. pseudocatenulatum* LI09 or *B. catenulatum* LI10 on the overall structural changes of gut microbial communities during D-GalN-induced acute liver injury. (**a**) The alpha diversity of the gut microbiome, determined by Simpson index and Chao1 index, in the LI09 or LI10 group was compared with that in the PC group. Values are expressed as the median with interquartile range. (**b**) The principal coordinate analysis (PCoA) plot shows the beta diversity of the gut microbiome with unweighted UniFrac distance derived from 16S-based sequencing data. Composition profiles of the caecal flora in the LI09 or LI10 group was compared with those in the PC and negative acute liver injury control (NC) groups.

The species diversity calculated by the Simpson diversity indices and the community richness determined by the Chao1 indices were similar between the PC group and the LI09 (p = 0.161, p = 0.332, respectively) or LI10 group (p = 0.161, p = 0.180, respectively) (Fig. 4a). Actually, the Simpson indices and Chao1 indices were also similar between the PC group and the LI06 or LI07 group. However, the Simpson index of LI08 group was higher than that of PC group, although the Chao1 indices were similar between these two groups (see Supplementary Fig. S3). An unweighted UniFrac principal coordinate analysis (PCoA) was used to analyse the overall structural changes of microbial communities (Fig. 4b). PC samples were roughly separated from NC samples (p = 0.013) along PC2, which explained 8.7% of the total variations. When the rats were administered with either *B. pseudocatenulatum* LI09 or *B. catenulatum* LI10 before D-GalN treatment, the gut microbiome was distinctly separated from that of the PC samples (p < 0.001, p = 0.015, respectively) and did not differ from that of the NC samples (p = 0.050, p = 0.607, respectively) along PC2, demonstrating that oral administration of *B. pseudocatenulatum* LI09 or *B. catenulatum* LI10 ameliorates the D-GalN-induced alterations of the gut microbiome.

**Figure 5:**
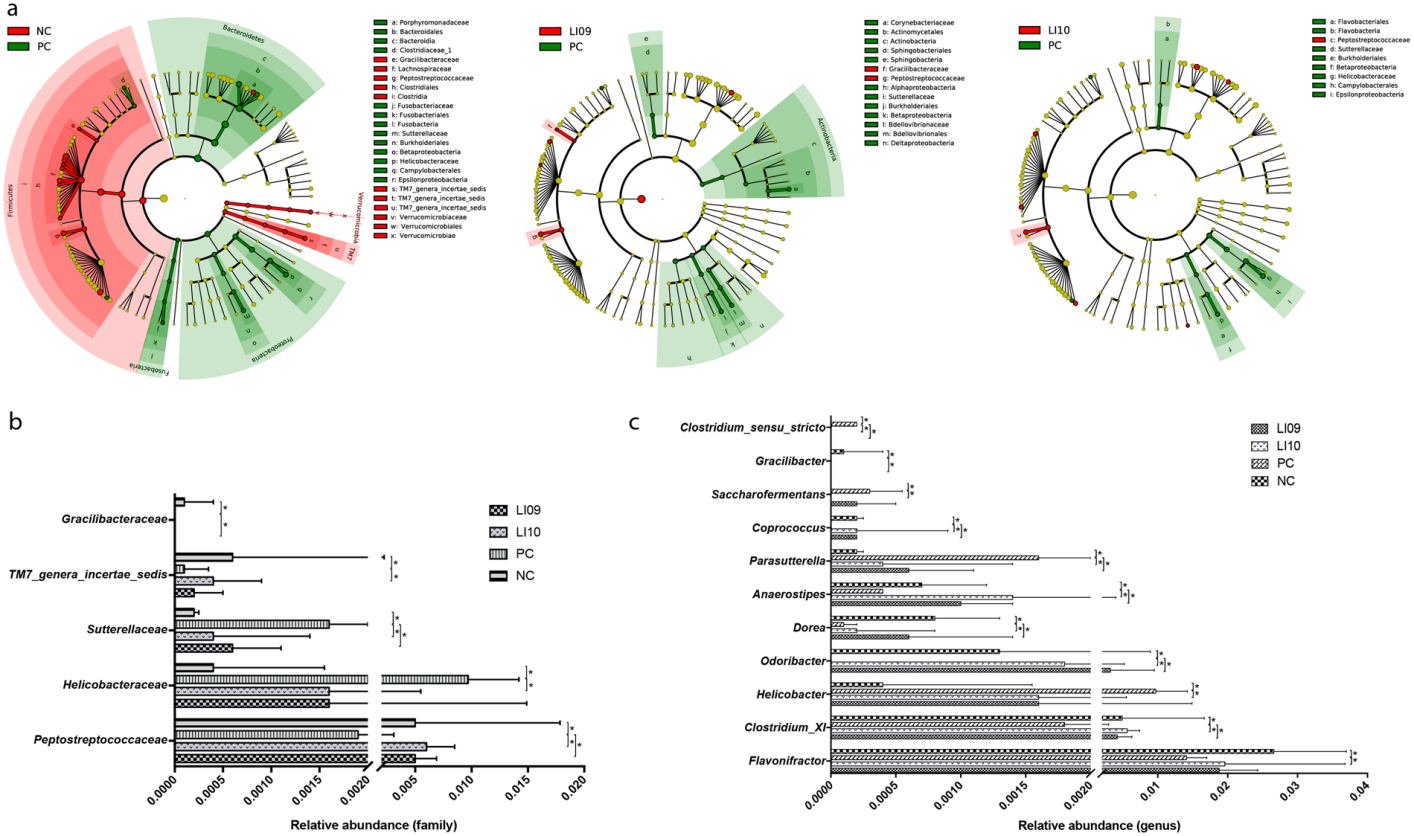
Effects of pretreatment with *B. pseudocatenulatum* LI09 or *B. catenulatum* LI10 on the alterations of gut bacterial taxonomic abundance during D-GalN-induced acute liver injury. (**a**) Bacterial taxa identified as differentially abundant between two groups analysed by linear discriminant analysis effect size (LEfSe). The bacterial taxa of the NC, LI09 and LI10 groups were compared with those of the PC group at different levels. Green indicates those bacterial taxa whose abundance were higher in the PC group, and red indicates those bacterial taxa whose abundance were higher in the other group. In addition, yellow indicates those bacterial taxa whose abundance show non-significant differences between the two groups. (**b**) The relative abundance at the bacterial family level in the LI09 or LI10 group was compared with that in the PC group. (**c**) The relative abundance at the bacterial genus level in the LI09 or LI10 group was compared with that in the PC group. Values are expressed as the median with interquartile range. *p < 0.05 compared with the PC group.

D-GalN-induced acute hepatic injury clearly affected the intestinal microbial structure at the phylum level (Fig. 5a). Compared with the NC group, the PC group demonstrated a marked depletion in Firmicutes, Verrucomicrobia and TM7 and a significant enrichment in Bacteroidetes, Proteobacteria and Fusobacteria. Oral *B. pseudocatenulatum* LI09 administration ameliorated the D-GalN-induced Proteobacteria enrichment, and administration of *B. catenulatum* LI10 alleviated the D-GalN-induced TM7 depletion.

Furthermore, acute liver injury had a much wider impact on the gut microbiome at the family and genus levels (Fig. 5). *Odoribacter*, which belongs to family *Porphyromonadaceae* of phylum Bacteroidetes, was depleted in the PC group, and administration of either *B. pseudocatenulatum* LI09 or *B. catenulatum* LI10 attenuated the D-GalN-induced *Odoribacter* depletion.

Administration of *B. pseudocatenulatum* LI09 or *B. catenulatum* LI10 contributed considerably to preventing the D-GalN-induced alterations in the abundance of Clostridiales of phylum Firmicutes. Specifically, genus *Clostridium_sensu_stricto*, the main component of family *Clostridiaceae_1*, was enriched in the PC group compared with that in the NC group, and administration of either *B. pseudocatenulatum* LI09 or *B. catenulatum* LI10 ameliorated its enrichment. Conversely, genus *Clostridium XI*, the main member of *Peptostreptococcaceae*, was depleted in the PC group, and this depletion was alleviated by pretreatment with either of these two strains. Only *B. pseudocatenulatum* LI09 attenuated the D-GalN-induced depletion of genus *Gracilibacter*. In addition, pretreatment with *B. pseudocatenulatum* LI09 or *B. catenulatum* LI10 also ameliorated the depletion of genera *Dorea*, *Anaerostipes* and *Coprococcus*, which all belong to family *Lachnospiraceae*. Although there was no difference in the abundance of family *Ruminococcaceae* between the PC and NC groups, genus *Saccharofermentans*, within this family, was enriched in the PC group; this enrichment was alleviated when the rats were pretreated with *B. catenulatum* LI10. Genus *Flavonifractor*, another *Ruminococcaceae* member, was depleted in the PC group, and treatment with *B. catenulatum* LI10 attenuated its depletion.

*B. pseudocatenulatum* LI09 or *B. catenulatum* LI10 administration significantly prevented the D-GalN-induced enrichment of opportunistic pathogens belonging to phylum Proteobacteria. *Parasutterella*, which was the primary contributor to the enrichment of class Betaproteobacteria, was enriched in the PC group, and administration of either *B. pseudocatenulatum* LI09 or *B. catenulatum* LI10 ameliorated its enrichment. In addition, the D-GalN-induced enrichment of another Proteobacteria member, genus *Helicobacter*, was attenuated in the group treated with *B. catenulatum* LI10.

By comparison, administration of none of the *B. longum* LI06, *B. longum* LI07 or *B. pseudocatenulatum* LI08 could alleviate the depletion of *Odoribacter*, *Clostridium XI*, *Dorea*, *Anaerostipes* and *Coprococcus* and the enrichment of *Clostridium_sensu_stricto* and *Parasutterella* simultaneously (see Supplementary Table S2).

### Correlations between involved inflammatory cytokines and gut bacterial genera were found in the groups treated with these two strains

**Figure 6:**
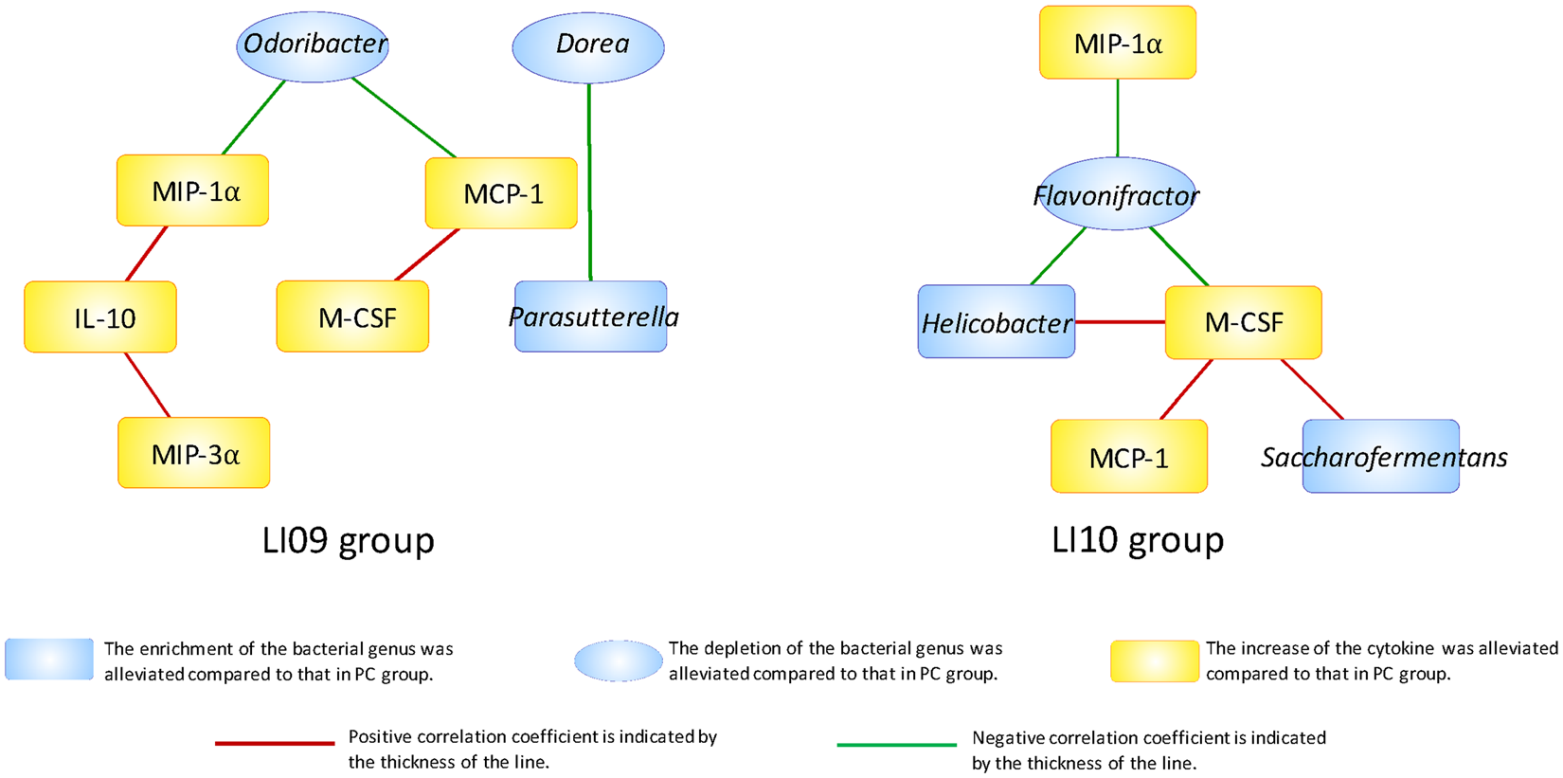
Correlation-network analysis of plasma cytokines and gut bacterial genera of D-GalN-sensitized rats who received *B. pseudocatenulatum* LI09 or *B. catenulatum* LI10. A Spearman correlation analysis was performed, and only correlations with p < 0.05 are displayed. Blue nodes represent bacterial genera, and yellow nodes represent cytokines. The ellipses indicate that the depletion of the bacterial genera was alleviated compared to that in the PC group, and the round rectangles indicate that the enrichment of the bacterial genera was alleviated or the increase of the cytokines was alleviated compared to that in the PC group. A red line connecting nodes represents positive correlation, and a green line represents negative correlation. Values of the corresponding correlation coefficients are indicated by the thickness of the line; the thicker the line, the greater the coefficient.

To verify that alleviating the alterations in the gut microbial composition plays an important role in reducing the increase in inflammatory cytokines, a systems biology approach (correlation-network analysis) was used to identify key linkages between the involved inflammatory cytokines and gut bacterial genera in acute liver injury (Fig. 6). In the *B. pseudocatenulatum* LI09-treated group, the chemokines MIP-1α and MCP-1 were negatively correlated with *Odoribacter*, while in the *B. catenulatum* LI10-treated group, the chemokine MIP-1α, along with M-CSF, was negatively correlated with *Flavonifractor*. Moreover, in both groups, a positive association was found between MCP-1 and M-CSF.

## Discussion

Acute liver failure, a severe form of liver damage caused by various factors, is an infrequent but life-threatening disease, with death occurring in up to 50% of the cases^18, 19^. The standard therapy for liver failure, liver transplantation, has improved the survival rate by approximately 40%; however, this option is strongly limited because of the shortage of donor livers^20, 21^. Thus, an artificial liver system (ALS), with the primary aim of detoxifying blood, is widely used in clinics until the patient’s own liver regenerates or a donor liver becomes available; nevertheless, the cost and safety of ALS remain a concern^22^. In this study, oral administration of *B. pseudocatenulatum* LI09 or *B. catenulatum* LI10 had extensive beneficial effects on induced liver damage in experimental rats, including reducing ALT, AST, glycylproline dipeptidyl aminopeptidase and total bile acid levels, as well as hepatic inflammation and necrosis, and attenuating systemic inflammatory responses, which could be of great importance in the prevention or adjuvant treatment of clinical acute liver failure in the future.

By contrast, administration of *B. longum* LI06 or *B. pseudocatenulatum* LI08 did not demonstrate significant protective effects on induced liver damage. And administration of *B. longum* LI07 showed beneficial effects only in the aspect of liver histology, which is a subjective indicator. Therefore, we didn’t consider *B. longum* LI07 as a candidate probiotics either.

Two strains from the same species, *B. pseudocatenulatum* LI08 and *B. pseudocatenulatum* LI09, demonstrated different effects. A similar situation was encountered by LX Lv *et al*.^23^, who reported that *Lactobacillus salivarius* LI01 were beneficial in the prevention of acute liver injury and *Lactobacillus salivarius* LI02 did not. *Lactobacillus rhamnosus* is another example. There are more than twenty kinds of strains of this species stored at American type culture collection (ATCC), but only the strain *Lactobacillus rhamnosus* GG (ATCC 53103) has been studied extensively on its various health benefits and become the world’s most studied probiotic bacterium. These conflicting findings may be explained by different sources of the strains which carry different genes and therefore have differences in their phenotypes.

There is increasing evidence suggesting that systemic inflammatory response syndrome (SIRS) occurs in acute liver failure through the release of damage-associated molecular patterns (DAMPs) and proinflammatory cytokines as a result of massive hepatic cell necrosis, which in turn plays a key role in the clinical course and outcome in acute liver failure patients^24^. In our study, strong inflammatory responses, as evidenced by significant increase in the levels of both proinflammatory cytokines (TNF- α, IL-1α, IL-1β, IL-2, and IL-6) and anti-inflammatory cytokine (IL-10), as well as chemokines (MCP-1, MIP-1α and MIP-3α) and colony-stimulating factor (M-CSF), were observed during acute liver injury progression. Lower levels of MCP-1, MIP-1α and M-CSF were seen at 24 h in both the LI09 and LI10 groups than in the PC group. MCP-1 and MIP-1α, both important members of the CC-chemokine family, recruit and activate monocytes/macrophages to the injured tissue area and regulate proinflammatory cytokines and adhesion molecules. They have been reported to play an important role in the early inflammatory response. M-CSF affects cells of the mononuclear phagocytic lineage in several ways, including regulating their survival, proliferation, and differentiation and as a major chemoattractant for these cells^25^. In the absence of M-CSF, monocytes could not be recruited to the sites of injured tissue due to the lack of MCP-1 synthesis^26^. Here, our data showed that administration of either *B. pseudocatenulatum* LI09 or *B. catenulatum* LI10 reduced the increased levels of MCP-1, MIP-1α and M-CSF (positively correlated with MCP-1), which may have led to the protection against liver injury, as suggested by the alleviation of the increase in serum ALT & AST and the amelioration of hepatic inflammation and necrosis.

The innate immune response is triggered by the recognition of translocated bacteria/bacterial products, followed by the release of chemokines and cytokines, leading eventually to bacterial killing^7^. In our study, bacterial translocation in the groups treated with these two strains was evaluated. MLNs are the first line of defence once the gut barrier has been breached. We found that the population of translocated bacteria to MLNs did decrease when rats were treated with either *B. pseudocatenulatum* LI09 or *B. catenulatum* LI10, which is consistent with the lower inflammatory response in these two groups.

Generally, only traces of gut-derived bacteria and their components can traffic in the portal vein to the liver due to the gut barrier, which is weakened upon the occurrence of liver disease. A disrupted intestinal barrier facilitates this translocation.

The integrity of the intestinal mucosa is one of the two important pillars that support the gut barrier function. In a liver disease situation, intestinal venous congestion will happen, followed by enterocyte necrosis or apoptosis, which manifest as damaged intestinal mucosal integrity. In this study, the incidence of subepithelial Gruenhagen’s space and epithelial lifting determined by H&E staining and the ruptured, sparse, and stunted microvilli of the intestinal epithelial cells determined by transmission electron microscopy indeed dropped in the LI09 and LI10 groups.

The intestinal colonization resistance, or the control of the growth of opportunistic microorganisms, is the other pillar. The normal flora acts as a barrier against colonization of potentially pathogenic microorganisms and against overgrowth of already present opportunistic microorganisms (mainly aerobic gram-negative bacteria)^27, 28^. The ecological balance of the gut flora is disrupted in hepatic injury, since bile is secreted abnormally and intestinal peristalsis slows down. Probiotic strains or their metabolites can modify the gut microbiological ecology. In our study, oral administration of *B. pseudocatenulatum* LI09 or *B. catenulatum* LI10 alleviated the D-GalN-induced alterations in the gut microbiome, as evidenced by the observation that the gut microbiome in samples pretreated with either *B. pseudocatenulatum* LI09 or *B. catenulatum* LI10 was significantly separated from that in PC samples and did not differ from that in NC samples in the PCoA analysis.

Bacterial phylotypes whose abundance was enriched in rats with acute liver damage were mostly associated with phylum Proteobacteria, including Betaproteobacteria and Epsilonproteobacteria. Genus *Parasutterella*, belonging to class Betaproteobacteria, was significantly enriched in PC samples. A high prevalence of these potentially pathogenic phylotypes has also been demonstrated in patients with Crohn’s disease^29^. In this study, administration of either *B. pseudocatenulatum* LI09 or *B. catenulatum* LI10 ameliorated the enrichment of genus *Parasutterella*, indicating the beneficial effects of these two strains. In addition, genus *Helicobacter*, belonging to class Epsilonproteobacteria, was also found to be significantly enriched in PC samples. The most widely known species of this genus is *Helicobacter pylori*, and some strains of this species, as well as several strains of non-*H. pylori Helicobacter*, are pathogenic to humans, as they are strongly associated with peptic ulcers, chronic gastritis and gastric cancer. The enrichment of genus *Helicobacter* was alleviated in the LI10 group.

Bacterial phylotypes with reduced abundance in rats with acute liver damage were strongly associated with class Clostridia (phylum Firmicutes), including *Lachnospiraceae*, *Peptostreptococcaceae* and *Gracilibacteraceae*, but not *Clostridiaceae_1*. *Lachnospiraceae* is known to participate in fibre fermentation in the human intestine^30^. The by-products of carbohydrate fermentation, such as butyrate, nourish cells lining the colon. In addition, these short-chain fatty acids (SCFA) seem to have anti-inflammatory effects by inducing regulatory T cells (Tregs) and, as a result, calibrate our immune system. The absence of these fermentation-related bacteria causes a decline in SCFA production and often correlates with diseases, such as asthma and inflammatory bowel disease^31^. Genera *Dorea*, *Anaerostipes* and *Coprococcus*, within family *Lachnospiraceae*, were all found to be depleted in the PC group. The latter two are both butyrate-producing bacteria. A decline of *Coprococcus* has been demonstrated in patients with intestinal^32, 33^, neuropsychological^34, 35^, infectious^36^, and atopic diseases^37^, as well as liver diseases^38–40^. A depletion of *Dorea* has been found in patients with intestinal^41^ and infectious diseases^36^, along with liver disease^42^, and a suppression of *Anaerostipes* has been shown in patients with paratuberculosis infection^43^. In addition, genus *Clostridium XI*, a SCFA-producing microorganism, within the family *Peptostreptococcaceae*, was also found to be significantly depleted in the PC group. The depletion of *Dorea*, *Anaerostipes, Coprococcus* and *Clostridium XI* in D-GalN-sensitized rats was attenuated by both the *B. pseudocatenulatum* LI09 and *B. catenulatum* LI10 strains, which further demonstrated the regulatory capacity of these two strains. Although there was no difference in the abundance of family *Ruminococcaceae* between the PC and NC groups, genus *Flavonifractor*, capable of cleaving the flavonoid C-ring^44^, within this family, was depleted in the PC group. A decline of a species belonging to *Flavonifractor* has also been demonstrated in obese individuals^45^. Treatment with *B. catenulatum* LI10 attenuated this depletion. Conversely, genus *Clostridium_sensu_stricto* (family *Clostridiaceae_1*), another member of class Clostridia, was found to be enriched in the PC group, which is consistent with some infant illnesses such as food allergies^46–48^ and necrotizing enterocolitis^49^. Its enrichment was ameliorated by either *B. pseudocatenulatum* LI09 or *B. catenulatum* LI10.

In addition, genus *Odoribacter*, another butyrate producer^50^, belonging to family *Porphyromonadaceae* (phylum Bacteroidetes), was also depleted in PC samples. The results of this study suggested that the abundance change of this genus in rats with acute liver injury is mostly accordance with metabolic syndrome^50, 51^. Its depletion was alleviated in both the LI09 and LI10 groups.

In short, these two strains both played a role in attenuating the depletion of health-promoting bacteria and ameliorating the enrichment of pathogenic bacteria, therefore partly maintaining the intestinal colonization resistance, which contributes to protecting the gut barrier function.

The regulation of the levels of MIP-1α and MCP-1 in the LI09 group and the levels of MIP-1α and M-CSF in the LI10 group might be associated with increased *Odoribacter* and *Flavonifractor* respectively, as evidenced by the significantly robust correlations, which are likely due to these two strains. As discussed above, the butyrate-producing genus *Odoribacter* and flavonoid-cleaving genus *Flavonifractor*, were both depleted in metabolic syndrome, which is closely associated with chronic low-grade inflammation. Seeing that MCP-1, MIP-1α and M-CSF are important chemoattractants for mononuclear phagocytic cells in the inflammatory response, it is indeed possible that *B. pseudocatenulatum* LI09 and *B. catenulatum* LI10 ultimately down-regulate the cytokines MCP-1, MIP-1α and M-CSF by modulating *Odoribacter* and *Flavonifractor* respectively. However, the underlying mechanisms will be possibly investigated in the future in view of the limited isolates and undeveloped culture techniques of *Odoribacter* and *Flavonifractor* at this stage.

In conclusion, our study shows the beneficial effects of *B. pseudocatenulatum* LI09 and *B. catenulatum* LI10 against liver injury induced by D-GalN, which might be mediated through the protection of the gut barrier function, specifically by alleviating intestinal flora dysbiosis and preventing intestinal epithelial cell damage. The restored gut barrier function may contribute to reducing bacterial translocation and finally down-regulating the overactive immune response. We will next evaluate their roles in attenuating liver damage in other experimental models of liver injury to facilitate the development of probiotic products in the future.

## Methods

### Strains and culture conditions

Five strains were used in this study: *B. longum* LI06 (CGMCC 10385), *B. longum* LI07 (CGMCC 10386), *B. pseudocatenulatum* LI08 (CGMCC 10387), *B. pseudocatenulatum* LI09 (CGMCC 10388), and *B. catenulatum* LI10 (CGMCC 10389). All strains were isolated from healthy volunteers, whose fecal samples obtained were immediately placed in an anaerobic environment before homogenized and plated on trypticase-phytone-yeast (QingDao RiShui, Ltd., Qingdao, China) agar. Five isolates were screened out through identifying with 16 s rDNA sequencing, stored at −80 °C and deposited in the China General Microbiological Culture Collection Center (CGMCC). The bacterial strains were revived following a standard approach (https://www.atcc.org/How_to_Revive_Cultures.aspx#bacteria2) and anaerobically cultured for 36 h at 37 °C. The cells were harvested by centrifugation at 6,000 g for 10 min. Subsequently, these cells were washed twice with normal saline and resuspended to a concentration of 3 × 10^9^ colony-forming units/ml in normal saline for further use.

### Animals and experimental design

Fifty-nine male Sprague-Dawley rats (from the Experimental Animal Center of Zhejiang Province, China), with an initial weight of 250–350 g, were randomized into seven groups with different treatments as follows: PC group (8 rats) and NC group (6 rats) both received 1 ml of physiologic saline by daily gavage; while LI06 group (9 rats), LI07 group (9 rats), LI08 group (9 rats), LI09 group (9 rats) and LI10 group (9 rats) received 1 ml (3 × 10^9^ colony-forming units/ml) of *B. longum* LI06, *B. longum* LI07, *B. pseudocatenulatum* LI08, *B. pseudocatenulatum* LI09 and *B. catenulatum* LI10 strains by daily gavage respectively. All animals were fed normal food (standard rat chow), kept at room temperature (22 °C) with a controlled 12-h light/dark cycle, and orally administered saline or bacteria through an oro-gastric tube once daily for 7 days. Acute liver injury was induced on the 8th day by an intraperitoneal injection of D-galactosamine (G0500, Sigma, Saint Louis, MO, USA) at a dose of 1.1 g/kg body weight in all groups except the NC group. All experimental procedures were performed in accordance with the 2011 National Institutes of Health Guide for the care and use of laboratory animals. The study protocol was approved by the Animal Care committee of Zhejiang University, China.

### Sample collections

Twenty-four hours after the induction of liver damage, the animals were anaesthetized and subjected to laparotomy through a large midline incision under aseptic conditions. Blood samples were collected from the inferior vena cava for liver function tests and to measure plasma cytokine levels. MLNs of the ileocaecal area were picked for bacterial translocation assay and caecal contents were obtained for gut bacterial microflora analysis. Tissue samples from the left lobe of the liver and ileum biopsied from a site approximately 2 cm away from the ileocaecal valve were harvested for histologic evaluation. All of the fifty-nine rats were analyzed for each following assay.

### Liver function tests

Blood samples were centrifuged at 3,000 g for 10 min to separate the serum for analysis. Concentrations of ALT, AST, glutamyltransferase, glycylproline dipeptidyl aminopeptidase, total bile acid, total bilirubin and albumin in the serum were quantified using a Hitachi 7600 automatic analyser according to the manufacturer’s instructions (Hitachi, Tokyo, Japan)^52^.

### Plasma cytokine analysis

Blood samples were centrifuged at 3,000 g for 10 min to separate the plasma and stored at −80 °C until analysis. The plasma (20 μl) was analysed using magnetic bead suspension arrays with the Bio-Plex Pro Rat Cytokine 24-Plex Panel (Bio-Rad) according to the manufacturer’s instructions. The 24-plex contains antibodies specific for erythropoietin, granulocyte colony-stimulating factor, granulocyte-macrophage colony-stimulating factor, growth-regulated oncogene-keratinocyte chemoattractant, IFN-gamma (γ), interleukin (IL)-1α, IL-1β, IL-2, IL-4, IL-5, IL-6, IL-7, IL-10, IL-12(p70), IL-13, IL-17A, IL-18, monocyte chemoattractant protein 1 (MCP-1), macrophage colony-stimulating factor (M-CSF), macrophage inflammatory protein 1 alpha (MIP-1α), MIP-3α, Regulated on Activation, Normal T Cell Expressed and Secreted, tumour necrosis factor alpha (TNF-α), and vascular endothelial growth factor. The samples were examined using a Bio-Plex 200 analyser, and the results were calculated using the Bio-Plex manager 6.0 software^53^. Values for granulocyte colony-stimulating factor, granulocyte-macrophage colony-stimulating factor and IFN-γ from most samples were below the detection limit, and these cytokines were excluded from the data analysis. Additionally, a few values for IL-1α, IL-2, IL-4, IL-6, IL-13, IL-17A, IL-18, erythropoietin, Regulated on Activation, Normal T Cell Expressed and Secreted and TNF-α were below the detectable limit. Since the standard curve lower limits of all these cytokines were above 0.1 pg/ml, and the statistical analysis was performed by rank sum test, those undetectable values were assumed to be 0.1 pg/ml for statistical purposes^54^.

### Bacterial translocation

A one-fold amount of sample from the MLNs was weighed and milled in a sterile glass homogenizer containing a nine-fold amount of physiological saline. Then, 50 μl of this homogenate (1/10 dilution) was plated on brain-heart infusion agar (BHI, Oxoid, Thermo Fisher Biochemicals Ltd., Beijing, China) in duplicate within 30 min of the sample collection and incubated for 72 h at 37 °C under either aerobic or anaerobic condition. Bacterial translocation was thought to occur if bacteria grew in the culture medium with the MLN homogenate. The number of colony-forming units from each plate was counted, and the number of translocated bacteria was expressed as CFU per gram of tissue.

### Histological evaluation

Tissue samples from the left lobe of the liver and the terminal ileum were immediately fixed in 10% neutral formalin when taken from the anesthetized rats and then embedded in paraffin, cut into 2-μm sections, stained with haematoxylin and eosin (H&E), and analysed by a pathologist who was blind to the groups. At least three slides were studied from each specimen.

The tissue damage of the liver was semiquantitatively assess by a histological score consisting of two categories from the Histological Activity Index^55^, intralobular degeneration and focal necrosis (score, 0–1 or 3–4) and portal inflammation (score, 0–1 or 3–4), which reflect the acute injury of livers. Intestinal mucosal lesions were classified as described by Chiu *et al*.^17^. Score 0 is defined as normal mucosa and score 1 is the development of subepithelial Gruenhagen’s space at the tip of the villus. This space is more extended in score 2. In score 3, there is a massive epithelial lifting down the side of the villus. And in score 4, the villus is denuded of epithelium. It is characterized by a loss of the villus itself in score 5.

### Electron microscopy

Ileal mucosal specimens fixed in 2.5% glutaraldehyde were post-fixed, dehydrated and embedded in epoxy resin. Ultrathin sections were made and post-stained in uranyl acetate and lead citrate^56^. The ileal mucosal ultrastructure was analysed on a Philips Tecnai 10 electron microscope (Philips, Eindhoven, the Netherlands). The length, diameter, linear density of microvilli was observed to learn the situation of microvilli damage^57^.

### 16 S rRNA sequencing

Caecal content samples (200 mg/aliquot) were immediately frozen upon collection from euthanized rats and stored at −80 °C before analysis. DNA extraction was performed using a QIAamp® Fast DNA Stool Mini Kit (QIAGEN, Hilden, Germany) according to the manufacturer’s instructions.

The bacterial 16 S rRNA V3–V4 regions were amplified by PCR using Phusion® High-Fidelity DNA Polymerase (New England Biolabs, Ipswich, MA, USA). The universal primer pairs were 319 F (5′-ACTCCTACGGGAGGCAGCAG-3′) and 806 R (5′-GGACTACHVGGGTWTCTAAT-3′). The reactions were hot started at 98 °C for 30 s, followed by 40 cycles of 98 °C for 10 s, 52 °C for 30 s, and 72 °C for 45 s, with a final extension step at 72 °C for 10 min. The amplified products from different samples were purified using the QIAquick Gel Extraction kit (QIAGEN, Hilden, Germany) and then mixed accordingly to achieve equal amounts in the final mixture. An amplicon library was constructed with a DNA sample preparation kit (Illumina, San Diego, CA).

Sequencing was conducted on an Illumina MiSeq platform (Illumina, San Diego, CA) according to the manufacturer′s instructions. Raw sequence reads were assigned to each sample according to their unique barcode pairs. Overlapping paired-end reads were merged to form tags using FLASH (version 1.2.8)^58^. The following quality control criteria were used: (1) an exact match to at least one end of barcodes and primers; (2) no undetermined bases in the tags; and (3) the number of mismatches in overlap region was no more than 3.

Operational taxonomic units (OTUs) were clustered with a 97% similarity cut-off using Usearch^59^. Alpha diversity was determined using the R program^60^ “rich” and “diversity” packages. Principal coordinate analysis (PCoA) as a standard multivariate statistical technique was performed with the R program “ade4” package to explain differences among microbial communities. The longest sequence in each OTU was chosen as the representative sequence for identification using the RDP (Ribosomal Database Project) database v.11.3^61^. Taxonomic ranks were assigned to each tag using RDP classifier v.2.10.1^62^, using 0.8 as the confidence coefficient.

### Statistical Analysis

A t-test (for those with normal distribution) or Mann–Whitney U test (for those with skewed distribution) was used to determine differences between two groups. The Spearman rank correlation coefficient was used for linear correlation analysis. The data were analysed using SPSS 20.0 and GraphPad Prism 7 and presented as the mean ± SD or median with interquartile range. A *p-value* < 0.05 derived from a two-tailed test was considered statistically significant. The network was presented after the correlation analysis using Cytoscape^63^.

## Electronic supplementary material

Supplementary Information 
