# Supplementary material for: Bifidobacterium pseudocatenulatum LI09 and Bifidobacterium catenulatum LI10 attenuate D-galactosamine-induced liver injury by modifying the gut microbiota
Source: Sci Rep. 2017 Aug 18;7:8770. doi: 10.1038/s41598-017-09395-8 (PMC5562910; doi:10.1038/s41598-017-09395-8)
Supplement: Supplementary file 1 — Supplementary Information [file 41598_2017_9395_MOESM1_ESM.pdf]

Bifidobacterium pseudocatenulatum LI09 and *Bifidobacterium catenulatum* LI10 attenuate D-galactosamine-induced liver injury by modifying the gut microbiota

Daiqiong Fang[1,2,+], Ding Shi[1,2,+], Longxian Lv[1,2,+], Silan Gu[1,2,+], Wenrui Wu[1,2,+], Yanfei Chen[1,2], Jing Guo[1,2], Ang Li[1,2], Xinjun Hu[1,2], Feifei Guo[1,2], Jianzhong Ye[1,2], Yating Li[1,2], Lanjian Li[1,2,*]

[1]State Key Laboratory for Diagnosis and Treatment of Infectious Diseases, The First Affiliated Hospital, College of Medicine, Zhejiang University, Hangzhou, Zhejiang, 310003, China

[2]Collaborative Innovation Center for Diagnosis and Treatment of Infectious Diseases, Hangzhou, 310003, China

*Corresponding author: ljli@zju.edu.cn

[+]These authors contributed equally to this work.

Supplementary materials

Supplementary Table 1. Effects of pretreatment with *Bifidobacterium longum* LI06, *Bifidobacterium longum* LI07 or *Bifidobacterium pseudocatenulatum* LI08 on bacterial translocation during D-GalN-induced acute liver injury

Group	MLN (log10 CFU/g)
LI06 (n=9)	3.2±0.3
LI07 (n=9)	2.8±1.4
LI08 (n=9)	2.7±1.8
PC (n=8)	3.7±0.5
NC (n=6)	2.8±0.3*

Values are expressed as the mean ± SD. *p<0.05 compared with the PC group.

Supplementary Table 2. Effects of pretreatment with *B. longum* LI06, *B. longum* LI07 or *B. pseudocatenulatum* LI08 on the alterations of gut bacterial abundance of family and genus levels during D-GalN-induced acute liver injury

	LI06 (n=9)	LI07 (n=9)	LI08 (n=9)	PC (n=8)	NC (n=6)
Family					
Peptostreptococcaceae	0.0024(0.0009,0.0068)	0.0018(0.0013,0.0055)	0.0048(0.0014,0.0078)	0.0019(0.0012,0.0031)	0.005(0.0044,0.0179)**
Helicobacteraceae	0.0008(0.0001,0.0211)	0.006(0.0015,0.0265)	0.0038(0.0016,0.0091)	0.0097(0.0059,0.0142)	0.0004(0.0002,0.0016)**
Sutterellaceae	0.0012(0.0004,0.0022)	0.0016(0.0014,0.0032)	0.0002(0,0.0016)*	0.0016(0.0009,0.002)	0.0002(0,0.0003)**
TM7_genera_incertae_sedis	0.0004(0.0001,0.0006)	0.0004(0.0002,0.0005)*	0.0002(0,0.0005)	0.0001(0,0.0004)	0.0006(0.0004,0.0023)**
Gracilibacteraceae	0(0,0.0001)	0(0,0.0002)*	0(0,0.0001)	0(0,0)	0.0001(0,0.0004)*
Genus					
Flavonifractor	0.016(0.01,0.0256)	0.0242(0.0167,0.0319)*	0.0154(0.0081,0.0194)	0.0141(0.0107,0.017)	0.0266(0.0147,0.037)*
Clostridium_XI	0.002(0.0005,0.0059)	0.0014(0.0012,0.0053)	0.0046(0.0008,0.0064)	0.0018(0.0011,0.003)	0.0049(0.0044,0.0167)**

Helicobacter	0.0008(0.0001,0.0211)	0.006(0.0015,0.0264)	0.0038(0.0016,0.0091)	0.0097(0.0059,0.0142)	0.0004(0.0002,0.0016)**
Odoribacter	0.0046(0.0022,0.0157)**	0.0038(0.0023,0.0103)***	0.0022(0.0019,0.0029)**	0(0,0.0013)	0.0013(0.0006,0.009)*
Dorea	0.0002(0,0.0002)	0.0002(0.0001,0.0004)	0(0,0.0003)	0.0001(0,0.0002)	0.0008(0.0002,0.0013)*
Anaerostipes	0.0008(0.0002,0.0019)	0.0012(0.0001,0.0022)	0.0004(0.0001,0.0024)	0.0004(0.0004,0.0006)	0.0007(0.0004,0.0012)*
Parasutterella	0.0012(0.0004,0.0022)	0.0016(0.0014,0.0032)	0.0002(0,0.0016)*	0.0016(0.0009,0.002)	0.0002(0,0.0003)**
Coprococcus	0.0004(0.0001,0.0006)**	0.0002(0,0.0005)*	0.0002(0.0002,0.0004)***	0(0,0)	0.0002(0.0002,0.0003)**
Saccharofermentans	0.0002(0,0.0007)	0(0,0.0004)	0.0002(0,0.0003)	0.0003(0.0001,0.0006)	0(0,0.0001)*
Gracilibacter	0(0,0.0001)	0(0,0.0002)*	0(0,0.0001)	0(0,0)	0.0001(0,0.0004)*
Clostridium_sensu_stricto	0(0,0.0001)*	0(0,0)**	0.0002(0,0.0005)	0.0002(0.0002,0.0004)	0(0,0)**

Values are expressed as the median with interquartile range. Compared with the PC group, *p<0.05, **p<0.01, ***p<0.001.

Supplementary Figure 1. Effects of pretreatment with *B. longum* LI06, *B. longum* LI07 or *B. pseudocatenulatum* LI08 on hypercytokinemia during D-GalN-induced acute liver injury

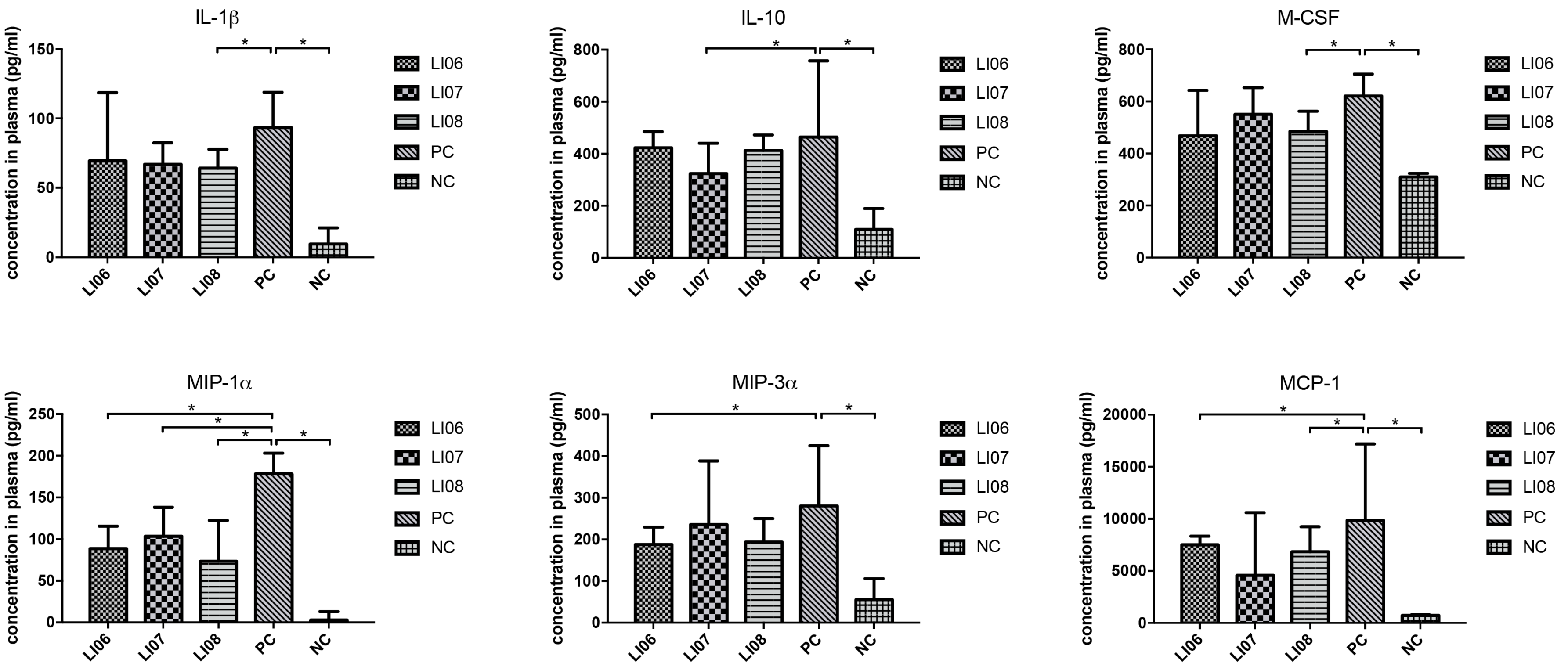

Values are expressed as the median with interquartile range. *p<0.05 compared with the PC group.

Supplementary Figure 2. Effects of pretreatment with *B. longum* LI06, *B. longum* LI07 or *B. pseudocatenulatum* LI08 on ileal histological abnormalities during D-GalN-induced acute liver injury

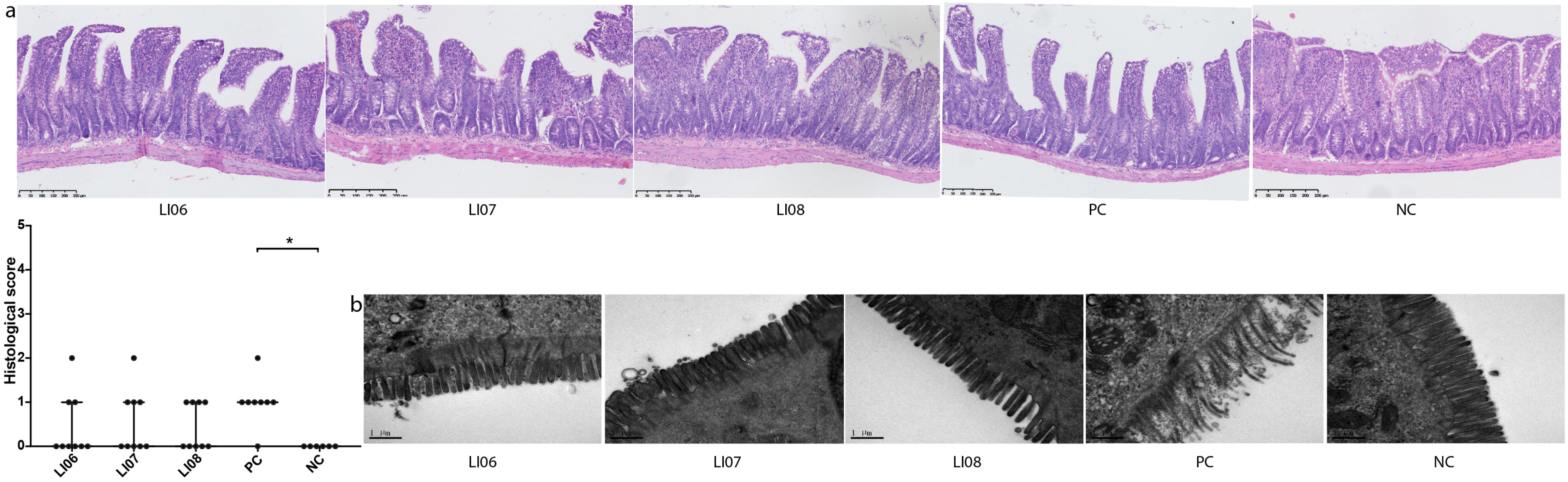

(a) Representative images of distal ileal H&E staining and histological scores of distal ileums based on these images. Values are expressed as the median with interquartile range. *p<0.05 compared with the PC group. (b) Representative electron microscopy images of the distal ileum. Microvilli of the intestinal epithelial cells were examined.

Supplementary Figure 3. Effects of pretreatment with *B. longum* LI06, *B. longum* LI07 or *B. pseudocatenulatum* LI08 on the changes of alpha diversity of gut microbial communities during D-GalN-induced acute liver injury

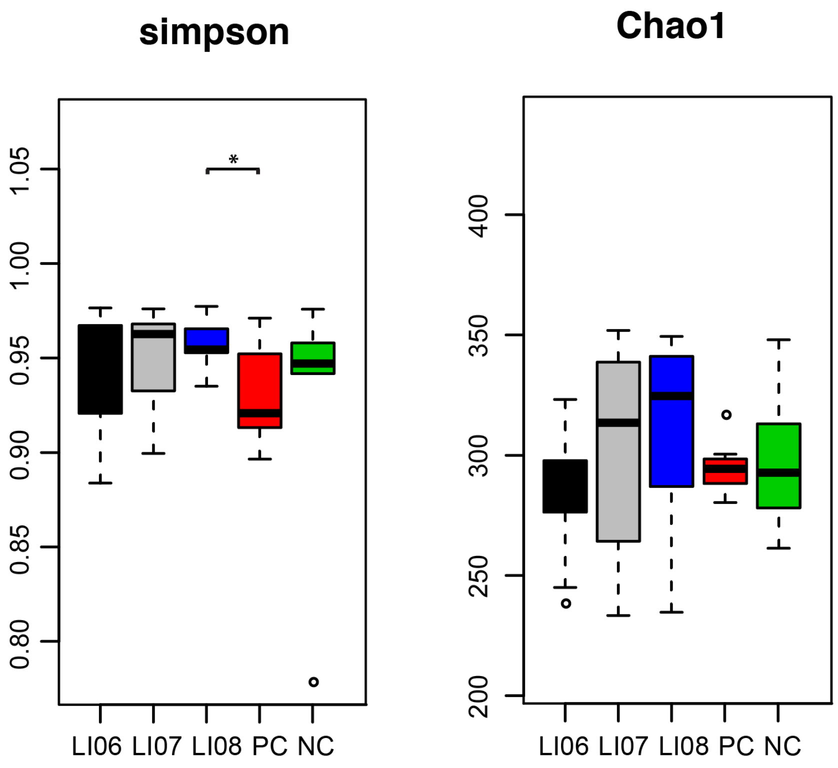

The alpha diversity of the gut microbiome, determined by Simpson index and Chao1 indices, in the LI06, LI07 or LI8 group was compared with that of the PC group. Values are expressed as the median with interquartile range. *p<0.05 compared with the PC group.
